# The Bergen–Yale Sexual Addiction Scale (BYSAS): Longitudinal Measurement Invariance Across a Two-Year Interval

**DOI:** 10.1007/s11126-024-10087-6

**Published:** 2024-08-29

**Authors:** Rapson Gomez, Taylor Brown, Vasileios Stavropoulos

**Affiliations:** 1 School of Health Sciences and Psychology, Federation University, Ballarat, Australia; 2 Applied Health, School of Health and Biomedical Science, RMIT University, 264 Plenty Rd, Mill Park, Melbourne, VIC 3082 Australia; 3 Department of Psychology, National and Kapodistrian University of Athens, Melbourne, Greece

**Keywords:** Sex addiction, The bergen–yale sexual addiction scale, BYSAS, Confirmatory factor analysis, Longitudinal measurement invariance, Ordinal data

## Abstract

The Bergen–Yale Sexual Addiction Scale (BYSAS; [1]) is arguably the most popular questionnaire at present for assessing sex addiction. Employing Confirmatory Factor Analysis (CFA) and treating item scores as ordered categorical, we applied Weighted Least Square Mean and Variance Adjusted Chi-Square (WLSMV) extraction to investigate the longitudinal measurement and structural invariance of ratings on the BYSAS among 276 adults (mean = 31.86 years; SD = 9.94 years; 71% male) over a two-year period, with ratings at three yearly intervals. Overall, there was support for configural invariance, full loading, full threshold, the full unique factor invariance; and all structural (latent variances and covariances) components. Additionally, there was no difference in latent mean scores across the three-time points. The psychometric and practical implications of the findings are discussed.

**Supplementary Information:**

The online version contains supplementary material available at 10.1007/s11126-024-10087-6.

The Bergen–Yale Sexual Addiction Scale (BYSAS) is a popular measure to assess sex addiction (i.e., frequent and persistent problematic sexual behavior; [1]). While previous research has demonstrated its unidimensionality and other psychometric properties, a notable gap in this area is the absence of data on its longitudinal measurement invariance, leaving its clinical and empirical utility uncertain. Thus, the current study aimed to examine the longitudinal measurement invariance of the BYSAS in a group of adults from the general community.

Recently, the International Classification of Diseases (ICD − 11) has marked a pivotal advancement by recognizing Problematic Sexual Behavior (PSB), which conceptually overlaps with sex addiction, placing it under the umbrella of impulse control disorders [2]. This classification, however, encompasses criteria characteristic of both addiction and compulsion [3], sparking nuanced debates on the nature of PSB as either primarily addictive [4–6], impulsive [7], or compulsive [8, 9], with some arguing that addiction, compulsivity, and impulsivity are not mutually exclusive. Moreover, the diversity in defining problematic sexual behaviors—often labeled as addictive—without consistently applying the six core components of addiction (i.e., preoccupation, tolerance, mood modification, withdrawal symptoms, relapse, functional impairment; [10, 11]) contributes to this complexity. This issue is further compounded by the absence of a corresponding diagnosis in the Diagnostic and Statistical Manual of Mental Disorders (DSM − 5; [12]), leading to empirical evidence that is often confounded by various definitions, criteria, and measurement tools [11].

In alignment with Griffiths’ components model of addiction [10, 13], the BYSAS was developed to encapsulate key aspects of sex addiction: salience, mood modification, tolerance, withdrawal symptoms, conflict, and relapse. Its establishment sought to offer a structured measure amid the ongoing debates and the evolving diagnostic landscape, where the formal recognition of sex addiction remains contentious within the psychiatric community. However, the ICD-11 has introduced Compulsive Sexual Behavior Disorder (CSBD) as a new diagnostic category, signifying a significant step in recognizing and classifying these behaviors. CSBD is described as a persistent pattern of failure to control intense, repetitive sexual impulses or urges leading to repetitive sexual behavior over an extended period, causing significant distress or impairment in key areas of functioning [3]. This inclusion follows extensive discussions on the conceptualization of compulsive sexual behaviors, underlining the need for a clear understanding and assessment of such behaviors. Despite the development of various scales to measure CSB, including the Compulsive Sexual Behavior Disorder Scale–CSBD-1 [14], the BYSAS’s unique focus on sex addiction through Griffiths’ [10] addiction components provides a valuable framework for assessing problematic sexual behavior within the context of this evolving diagnostic landscape.

In the initial scale development and validation study of the BYSAS [1], the BYSAS ratings were obtained from 23,533 participants in Norway. Exploratory factor analysis (EFA) involving one-half of the total sample supported a one-factor solution. However, there was local dependence between items 1 and 2. For the other half of the sample, confirmatory factor analysis (CFA) with a weighted least square estimation of a revised model, incorporating error covariances between items 1 and 2, was supported. The BYSAS factor showed good internal consistency reliability and external (convergent and discriminant) validity, exhibiting positive correlations with extroversion, neuroticism, intellect/imagination, and narcissism; and negative correlations with conscientiousness, agreeableness, and self-esteem.

To date, a number of studies have expanded the initial exploration of the psychometric properties of the BYSAS [14–17]. In a study involving 177 Israeli males, Paz et al. [15] reported that principal component analysis (PCA) supported a one-factor model, while CFA (*N* = 92) with maximum likelihood estimation of the originally proposed one-factor BYSAS model revealed poor fit. A revised model, including error covariances for item 1 with items 4, 3 and 4, and item 4 with item 5 showed a good fit. Youseflu et al. [17] examine the support for the originally proposed one-factor BYSAS model in a group of 364 Iranians. They reported that both the EFA (*N* = 364) and CFA (*N* = 380) with weighted least squares (WLS) extraction supported this model. Similarly, in a more recent study involving 1230 Italians, Soraci et al. [16] reported that the CFA with diagonal weighted least squares estimation (DWLS) supported the one-factor model. Zarate et al. [18], examining item response theory properties, supported the original one-factor model using CFA with DWLS extraction, with participants from their study included in the current study. The participants in that study were participants recruited at Time 1 (*N* = 1097) in the current study. In summary, studies using extraction methods suitable for categorical or ordinal data, such as WLS or DWLS, have consistently supported the original one-factor model. Additionally, all cited factor analysis studies demonstrated good psychometric properties, including internal consistency reliability, test-retest reliability (over two weeks), convergent and divergent validity, and measurement invariance across gender. Notwithstanding this, there has been little empirical attention to longitudinal measurement invariance.

In the context of measurement invariance across time, longitudinal measurement invariance ensures that reporting the same latent score at different time points (say Time 1, Time 2, and Time 3), corresponds to endorsing the same observed rating scores at those different time points (i.e., Time 1, Time 2, and Time 3; [19]). The opposite is true when there is no support for measurement invariance. Weak or no support for longitudinal measurement invariance suggests that the ratings at the different time points cannot be justifiably compared due to the potential confounding by different measurement and scaling properties. Therefore, for creditable comparison of ratings at various time points, empirical information on measurement invariance of the BYSAS items is required. Support for longitudinal measurement invariance is theoretically and clinically important as this is necessary for accurately tracking the developmental trajectory of sex addiction symptoms, assessing the effectiveness of clinical treatments over time, and increasing the generalizability of findings based on BYSAS data collected longitudinally. Therefore, the absence of such data can be considered a major limitation, both clinically and empirically, in the psychometric properties of the BYSAS.

The study by Youseflu et al. [17] supported test-retest reliability over a two-week interval, however, it is crucial to distinguish this from longitudinal (or test-retest) invariances.

While longitudinal invariance evaluates if the same observed scores across time for a measure reflects the same levels of the underlying latent trait scores, test-retest reliability evaluates if the scores on a measure obtained across two or more time points are stable (such as demonstrating high correlation) over time (see [20]). Indeed, demonstrating test-retest invariance is a prerequisite for establishing test-retest reliability, an aspect not yet explored for the BYSAS.

The application of CFA procedures for measuring measurement invariance (including longitudinal measurement invariance) involves comparing progressively more constrained models that test several levels of invariance. In the context of longitudinal measurement invariance, this involves showing that (i) the latent factor structure remains the same between time points (baseline or configural invariance); (ii) the associations/strengths of like items with their latent factors are the same at different time points (metric or loading invariance); (iii) the item intercepts (for continuous scores) or threshold (for ordered categorical scores) of like items are the same at different time points (scalar or intercept/threshold invariance); and (iv) the item uniqueness variances of like items are the same at different time points (uniqueness or the unique factor invariance). For structural invariance, this involves confirming that the variances and covariances of similar latent factors remains the same at different time points (variances and covariances invariance).

## Aim of the Study

Given existing limitations, the current study aimed to examine longitudinal measurement invariance of BYSAS ratings in a group of adults from the general community. Longitudinal measurement invariance was examined over a two-year interval, with ratings at three-time points (i.e., 2021, 2022, and 2023). As this examination was primarily exploratory, no particular expectations have been generated.

## Method

### Participants

Participants were from the general community and constituted a normative online convenience sample. In terms of individuals with usable scores, at Time 1 there were 968 English-speaking adults, and at Time 2 it comprised of 462 adults who participated at Time 1 at Time 3, 276 adults participated in both Time 1 and Time 2. Therefore, the participation rate from Time 1 to Time 2 was 47.7% (462/968), from Time 2 to Time 3 was 59.9% (276/462), and from Time 1 to Time 3 was 28.5 (276/968).

To detect attrition bias in the characteristics of the final sample, *t*-tests were employed to compare those subjects who responded to all waves of the study (i.e., participants in Time 3) with those who dropped out from one wave to another [21]. For this, we used the mean scores for BYSAS total scores at Time points 1 and 2 as the dependent variable. Significant differences (between participants in Time 3 and respondents in Time 1 who did not respond in Time 3; and between participants in Time 3 and respondents in Time 2 who did not respond in Time 3) for the BYSAS total scores can be interpreted as congruent with attrition bias, whereas non-significant differences can be interpreted as not congruent with attrition bias. Supplementary Table S1 shows the descriptives for the variables that tested attrition bias, and the results of the *t-*test comparisons. As shown for both Time 1 and Time 2, the relevant groups did not differ for BYSAS total scores. These findings can be interpreted in terms of the data at Time 3 as unlikely to be impacted by attrition bias. Considering the likelihood that there was no attrition bias, only the 276 participants who completed ratings for all time points were used in this study.

Soper’s [22] software for computing sample size requirements for CFA models was used to evaluate the sample size requirement for the present study. For this, the anticipated effect size was set at 0.3, power at 0.8, the number of latent variables at 3 (covering the three-time points), the number of observed variables at 18 (covering the three-time points), and probability at 0.05. The analysis recommended a minimum sample size of 200. Our sample size (*N* = 276) exceeds this recommendation.

Table 1 provides background information on the 276 participants involved in the study. As shown, their ages ranged from 18 years to 62 years (mean = 31.86 years; *SD* = 9.94 years) and included 196 men (71%; mean age = 31.92 years, *SD* = 10.84 years), and 75 women (32.9%; mean age = 32.12 years, *SD* = 10.84 years). Additionally, five individuals (1.8%) did not identify their gender. No significant age difference was found across men and women, *t* (269) = 0.15, *p* = 0.88. In terms of sociodemographic background, slightly more than half the number of participants (66.5%) reported being employed, and most of them reported having completed at least secondary education (97%). Racially, most of the participants identified themselves as “white” (69.2%), and slightly less than half the number of participants (42.4%) indicated that they were involved in some sort of romantic relationship. Overall, the sample comprised of mainly online employed white educated males, with at least secondary school education.

**Table 1:** Frequencies and Descriptive Statistics of Background Variables Collected in the study

Variables	Frequencies/descriptive statistics
Number of participants	Frequency = 276
Age (Time 1), years	Mean (*SD*); range: 31.86 (9.94); 18–62
Sex	Frequency (percentage): Women = 75(27.2%); Men = 196(71%); 5 (1.8%)
Age by gender, years; ∆men and women	Mean (*SD*): women = 32.12 (10.84); men 31.92 (9.66); *t* (269) = 0.15, *p* = 0.88
Employed	Frequency (percentage) = 183 (66.5%)
*Race*	
White	Frequency (percentage) = 191 (69.2%)
Black/African American	Frequency (percentage) = 19 (6.9%)
Asian	Frequency (percentage) = 51 (18.5%)
Hispanic/Latino	Frequency (percentage) = 11 (4.0)
Others (Aboriginal, Pacific Islander, Mixed)	Frequency (percentage) = 4 (1.5%)
*Highest educational level*	
Primary	Frequency (percentage) = 3 (1.1%)
Secondary	Frequency (percentage) = 71 (25.7%)
Technical	Frequency (percentage) = 30 (10.9%)
Some University	Frequency (percentage) = 130 (59.1%)
Others	Frequency (percentage) = 5 (1.8%)
*Relationship*	
Is involved	Frequency (percentage) = 117 (42.4%)

Note *SD* = Standard Deviation; Max/Min = Maximum/Minimum

### Measures

All individuals who participated in the study provided demographic information (via online questions), including their age, gender, ethnicity, highest education level completed, employment status, relationship status, and video game use and involvement. These were collected at the start of the study (Time 1). They also completed ratings of the BYSAS [1], and other measures. These ratings were obtained at three different time intervals, one year apart (in 2021, 2022, and 2023). Among the measures completed, only the BYSAS that measures symptoms associated with sex addiction are of relevance in this study.

The BYSAS includes six items with a time reference for the past year. It is a unidimensional measure. An example item is: “Spent a lot of time thinking about sex/ masturbation or planned sex?”. Items are responded to on a five-point scale (0 = very rarely, 1 = rarely, 2 = sometimes, 3 = often, and 4 = very often). Thus, the total score ranged from 0 to 24, with higher symptom scores indicating higher symptom severity. Based on ratings of the BYSAS [1], are classified addiction as follows: sex addict = at least four items endorsed as present [i.e., rated 3 (often) or 4 (very often)]; moderate sex addiction risk = total score ≥ 7 but not fulfilling the criteria for sex addiction; low sex addiction risk (total score between 1 and 6); and no sex addiction (total score of zero). The internal reliability for the BYSAS instrument was very good in the present study (Cronbach α = 0.83, 0.86, and 0.83 for Time 1, Time 2, and Time 3, respectively).

### Procedure

The study was approved by the Human Ethics Research Committee, (approving University masked for blind review). The study was advertised using both non-electronic and electronic (i.e., email and social media) methods. All data were conducted online through a web-based survey, assessing a range of addiction behaviors, personality, psychopathology, and coping. Time 1 data was collected between August 2019 and August 2020. Participants were invited to register their interest in the study via a Qualtrics link available on social media (i.e., Facebook; Instagram; Twitter), the (University name masked for blind review) websites and digital forums (i.e., reddit.com). The link took them to the Plain Language Information Statement (PLIS). Interested participants agreed to informed consent by clicking a button, followed by answering sociodemographic and internet gaming questions and relevant questionnaires (specifically, only the BYSAS is relevant to this study). Participants completed the survey on a computer at a location of their choice. After completing this step, participants were requested to voluntarily provide their email address to be included in prospective data collection wave(s), and to digitally sign the study consent form (box ticking).

Twelve months later (between August 2021 and August 2022), those who consented were contacted via email for voluntary participation in the survey’s second wave, which mirrored the initial survey components (PLIS, email provision, consent form, and survey questions). A total of 462 participated in the second data collection wave. A comparable procedure used in 2022 was used for collecting wave 3 data in 2023. The inclusion criteria included being over the age of 18 years old and participating in any kind of online activities, including but not limited to online gaming. The exclusion criteria included disqualifying only those responses that were incomplete or invalid. Due to the inclusion of questionnaires addressing one’s level of distress, those who had a current untreated severe mental illness were instructed (also included in the plain language information statement) not to participate to avoid any unforeseen/indirect emotional impact. Outside of these specified conditions, no additional criteria for inclusion or exclusion were imposed, allowing for a diverse and wide-ranging sample of participants.

### Statistical Procedures

Descriptives statistics, including mean and standard deviation scores, and dispersion (skewness, kurtosis, and Shapiro-Wilk) statistics were initially examined for all BYSAS items across all three points. According to Mishra et al. [23], an absolute skewness value ≥ 2 or an absolute kurtosis ≥ 4 may be used as reference values for determining considerable nonnormality (see also [24]).

All Confirmatory Factor Analysis (CFA) models were conducted using M*plus* (Version 7) software [25]. As will be evident by now, there are five response categories for all the six items in the BYSAS uses. Although these scores are ordinal, they can be treated as continuous as there are five response options [26, 27]. Due to pronounced nonnormality in the ratings for the BYSAS items (refer to Supplementary Table S2), coupled with the underrepresentation of response category 5 [26, 27], our data was treated as ordinal. This decision was further influenced by previous studies demonstrating that the endorsement of the originally proposed one-factor model was more apparent when data was treated as categorical or ordinal, with the WLS or DWLS (called WLSMV in M*plus*) estimator used as the extraction method. Consequently, the weighted least square mean and variance adjusted chi-square (WLSMV) estimator was employed in all CFA analyses. Recognized for its distribution-free and robust qualities, particularly suitable for ordered-categorical scores, the WLSMV estimator corrects for non-normality in the dataset [25]. This choice was favored over WLS estimation, which requires larger sample sizes and is more computationally intensive [28].

Prior to the tests for longitudinal measurement invariance, one-factor BYSAS models were assessed for fit separately at each time point (Time 1, Time 2, and Time 3). For these models, the ratings for all six items were loaded on a single latent factor, and the error variances were not correlated. Also, for identification, the variances for the first items were fixed to one.

In assessing longitudinal measurement invariance of the BYSAS scores across three-time points, the analysis initiated with a configural invariance model to ensure a consistent factor structure over time. Subsequently, evaluation escalated to metric/loading invariance by constraining the factor loadings across time points to be equal, thus verifying the constancy of item-factor relationships. Scalar/threshold invariance was then tested to ensure uniform item thresholds across the time intervals, essential for comparing latent traits. Following this, uniqueness/residual invariance was assessed to confirm equal measurement errors across time points. Structural invariance regarding latent factor variances and covariances was also examined, affirming stability in the relationships and variability of latent constructs over time. These invariance levels were sequentially validated using the WLSMV estimator in *Mplus*, ensuring that each model’s fit did not significantly deteriorate compared to its less constrained predecessor. This hierarchical testing approach, leveraging the robustness of the WLSMV estimator, enabled a rigorous examination of the BYSAS’s longitudinal measurement invariance, affirming its utility for reliable comparison across time points.

With reference to longitudinal measurement invariance, as the ratings for the BYSAS at Time 1, Time 2, and Time 3 lacked independence, multiple group CFA (usually applied for testing measurement invariance) could not be used. Rather, an extended single-group CFA model that included the ratings at all time points was used. Supplementary Fig. 1 shows the path diagram for evaluating longitudinal measurement invariance across the three-time points (Time 1, Time 2, and Time 3) for the BYSAS. As shown in the figure, the model combines the unidimensional factor model for Time 1, Time 2, and Time 3. However, the models at each of the time points are connected, with all like error variances and latent factors at the three time points being correlated with each other. For this study, we used the CFA approach illustrated by Liu et al. [29] for ordered-categorical indicators to examine longitudinal invariance. The WLSMV estimator in M*plus* and theta parameterization were utilized for testing these invariance models. We tested and compared sequentially the four hierarchical models, described earlier (see [29]), i.e., the configural invariance model, the loading invariance model, the threshold invariance model, and the unique factor invariance model. Loading, threshold, and unique factor invariance models parallel weak, strong, and strict measurement invariance [29]. The parameterization and M*plus* codes for these models are described in detail by Liu et al. [29]. Notwithstanding this, a brief description of this is provided in Supplementary Table S3.

At each level of invariance, full measurement invariance is inferred if the fit of the model does not differ from the previous model. When full measurement invariance is not found, the non-invariance items can be determined by freeing the equality constraints of the relevant parameter (for example, the threshold in the case of the scalar invariance model) at the relevant time points. This is done sequentially, beginning with the constrained parameter with the highest modification index (MI) until obtaining a final partial invariance model. The final partial invariance model is the model that does not differ from the previous model.

When there is support for full or partial measurement invariance, invariances for the structural components of the model (factor variance and covariance) can be examined. Although not related to structural invariance, equivalency for latent factor scores can be examined, taking into consideration non-invariance for the measurement model. Using the threshold invariance model, for testing the latent variance invariance model (M5), all latent variances in M3 are constrained equally across the time points; and in the latent covariance invariance model (M6), all latent covariances in M3 are constrained equally across the time points. In testing differences in latent mean scores, all latent mean scores in M3 are constrained equally across the time points. Again, at each step described above, the relevant invariance is inferred if the fit of the model does not differ from M3.

For examining the goodness-of-fit of the CFA models, the WLSMVχ^2^ was utilized. Similar to other χ^2^ values, large sample sizes lead to WLSMVχ^2^ values being exaggerated. As well as providing the WLSMVχ^2^, M*plus* also makes available approximate (or practical) fit indices. These include the root mean squared error of approximation (RMSEA), the comparative fit index (CFI), and the Tucker-Lewis Index (TLI). The current study employed these to assess the goodness-of-fit of models. For these fit indices, Hu and Bentler’s [30] recommendations were adopted: RMSEA values of 0.06 or below indicate good fit, 0.07 − 0.08 moderate fit, 0.08 to 0.10 marginal fit, and > 0.10 poor fit. Values of 0.95 > signified good model-data fit, and values of 0.90 and < 0.95 were taken as acceptable fit for the CFI and TLI. For comparing the various nested CFA models, we used the χ^2^ difference test (DIFF test). The differences in the approximate fit indices (CFI and RMSEA) were not considered for this purpose as this is not recommended for invariance testing when the WLSMV estimator is involved [29, 31]. For CFA models using WLSMV, Type I error rates could be inflated [31]. Examination of local fit indices, such as residuals and/or modification indices, is generally used to evaluate this.

As mentioned earlier, all data was collected online. Despite the current popularity of online data collection, some researchers have questioned the validity of the data collected in this manner (e.g., [32, 33]). Thus, we examined the potential for invalid data in our data set. While there are numerous methods for detecting this, we used a consistency check approach. In this approach, the consistency of responses across comparable items in the data set is evaluated [34]. In general responses across comparable items in the data set should be expected. If found, then it can be assumed that there is little or no invalid data collection problem. On the other hand, if the responses across comparable items differ noticeably, it can be interpreted as indicative of the possibility of invalid data collection problems. We check for invalid data in our data set via descriptive and dispersion statistics across the three-time points. These statistics are provided in Supplementary Table S2. As can be noticed the mean, SD, skewness, kurtosis, and Shapiro-Wilk values were highly comparable across the time points. Therefore, these findings can be interpreted as not indicating problems related to invalid data collection.

### Data Availability

Data used in the analysis in this study is available at request from the corresponding author.

## Results

### Missing Values and Descriptives (Mean and Standard Deviation)

As shown in Supplementary Table S2, for those participants involved in the analyses, the scores for only two items were missing at Time point 1. There was no missing value at Time points 2 and 3. The item mean scores suggest that generally the items were rated within 0 (very rarely) and 2 (sometimes), thereby indicating relatively low to moderate levels of sexual addiction for the group as a whole. Indeed, based on the same criteria used by Andreassen et al. [1] for classifying addiction, we found sex addict = 17.0%; moderate sex addiction risk = 19.2%; low sex addiction risk = 48.7%; and no sex addiction = 14.9%.

### Dispersion Statistics

In relation to dispersion (skewness, and kurtosis) statistics, across all three time points, the skewness values ranged from 0.13 to 3.83, with three values having values above ≥ 2. They all involved item 6. For kurtosis, they ranged from − 0.92 to 19.55, with three values having values above ≥ 4. They all involved item 6. Thus, several items had considerable nonnormality. Consistent with this interpretation, the Shapiro-Wilk values for all items at all time points were highly significant (*p* < 0.001).

### Fit of the One-Factor BYSAS Model at Time Points 1, 2 and 3

Table 2 displays the results of the fit for the one-factor BYSAS model at Time points 1, 2, and 3. As shown, based on Hu and Bentler’s [30] recommendations, at all three-time points, the CFI and TLI showed a good fit. The RMESA at Time 1 indicated adequate fit, and for Time 2 and Time 3, it indicated poor fit. Overall, these findings can be interpreted as indicating a mixed but sufficient fit for the one-factor BYSAS model at all three-time points. However, as the RMSEA showed questionable fit, we examined local misfit (residual and modification indices).

**Table 2:** Fit values for the BYSAS 1-factor CFA Model at Time 1, Time 2 and Time 3

Time point	χ^2^ (df)	CFI	TLI	WRMR	RMSEA (90% CI)
Time 1	32.27 (9)	0.99	0.98	0.69	0.1 (0.062 − 0.134)
Time 2	49.49 (9)	0.98	0.97	0.99	0.12 (0.094 − 0.164)
Time 3	43.84 (9)	0.98	0.97	0.88	0.12 (0.085 − 0.155)

Note CI = confidence interval; RMSEA = root mean square error of approximation; CFI = comparative fit index; TLI = Tucker-Lewis Index; WRMR = weighted root mean square residual. All WLSMV*χ*^2^ values were significant

The modification indices did not show any local misfit. The residuals, which represent the differences between the observed covariances/correlations and the model-implied covariances/correlation, showed some evidence of misspecification. Generally, correlation residuals > 0.10 are used to indicate the source of the model mis specified [35]. The findings at Time 1, indicated a residual correlation of ≥ 0.10 between item 1 and item 6. For Time 2, there was a residual correlation of ≥ 0.10 for item 1 with items 2 and 6; item 2 with items 5 and 6, and item 5 with item 6. For Time 3, there was a residual correlation of ≥ 0.10 for item 1 with items 3 and 6; and item 2 and item 6. Thus, at each time point, the relevant items in these relations may not be represented well in the one-factor BYSAS models. As will be noticed, the item that appears to be especially problematic at all time points is item 6. This is the same item that showed high skewness and kurtosis values at all time points.

Overall, while these findings indicate some degree of local misfit for the one-factor BYSAS model at all three-time points, it can be interpreted that at the global level, there was sufficient fit at all three time points to peruse our next goal of testing longitudinal measurement invariance across time.

### Longitudinal Measurement Invariance for the BYSAS 1-Factor CFA Model Across Time 1, Time 2, and Time 3

Table 3 presents an overall summary of the results for testing longitudinal measurement invariance for the BYSAS 1-factor CFA model across Time 1, Time 2, and Time 3. As shown, for the configural invariance model (M1), the CFI and TLI indicate an acceptable fit, and the RSMEA indicates a good fit. Thus, there was support for the configural invariance model. When we examined the residuals of the configural invariance model, we found that virtually all the residuals (138 or approximately 90%) were less than 0.10, thereby showing little evidence of local strain. Also, the residuals and modification indices indicated that constraining the thresholds of some indicators to be invariant across measurement occasions was not problematic, thereby suggesting that type 1 error rates were not inflated in our study.

**Table 3:** Results of the test for Longitudinal Measurement Invariance for the BYSAS 1-factor CFA Model Across Time 1, Time 2, and Time 3 using the WLSMV estimation with robust correction in Mplus

		Robust χ^2^ fit	Approximate Fit			DIFF Test			
#	Model (M)	WLSMV*χ*^2^ (*df*)	CFI	TLI	RMSEA (90% CI)	∆M	∆*df*	∆WLSMV *χ*^2^	*p* value
1	Configural	232.34 (114)	0.98	0.97	0.06 (0.05 − 0.07)				
2	Loading - M1 with all loadings free	231.5 (124)	0.98	0.98	0.056 (0.05 − 0.07)	M2 - M1	10	2.67	0.98
3	Threshold– M2 with all thresholds free	260.43 (158)	0.98	98	0.05 (0.04 − 0.06)	M3– M2	34	32.22	0.56
4	Unique factor invariance - M3 with all factor variance free	271.01 (170)	0.98	0.98	0.05 (0.04 − 0.06)	M4– M3	46	53.12	0.22
5	Factor variance invariance– M3 with all factor variance set equal	251.09 (161)	0.98	0.99	0.05 (0.03 − 0.06)	M5– M3	3	1.76	0.62
6	Factor covariance invariance– M3 with all factor covariance set equal	260.64 (162)	0.98	0.98	0.07 (0.06 − 0.08)	M6– M3	4	7.65	0.11
7	Factor mean invariance– M3 with all factor mean set equal	259.24 (159)	0.98	0.98	0.05 (0.04 − 0.06)	M7– M3	1	0.47	0.49

Note CI = confidence interval; *df* = degrees of freedom; RMSEA = root mean square error of approximation; CFI = comparative fit index; TLI = Tucker-Lewis Index. All WLSMV*χ*^2^ values were significant (*p* < 001)

Table 3 also shows that there is no difference between the full loading invariance model (M2) and the configural invariance model (M1), thereby indicating invariance for all factor loadings. The next analysis indicated no difference between the M3 model and the full loading invariance model (M2), thereby indicating invariance for all thresholds. The next analysis indicated no difference between the full loading invariance model (M2) and the unique factor invariance model (M4), thereby indicating support for full unique factor invariance.

In relation to structural invariance, there was no difference between the factor variances invariance (M5) and threshold variance invariance (M3) model; and there was no difference between the factor covariances invariance model (M6) and factor threshold invariance model (M3). Thus, there was invariance for all factor variances and covariances. Additionally, there was no difference for latent mean scores (M7) at Time 1, Time 2, and Time 3.

Therefore, overall, there was support for full measurement and structural invariance model. Additionally, there was no difference for latent mean scores over the two-year interval. Considering this, for brevity, we have presented in Table 4 the factor loadings, thresholds, and unique factor invariance for the BYSAS one-factor CFA configural model at Time 1 only.

**Table 4:** Unstandardized estimates of the parameter in the Longitudinal Configural Measurement Invariance Model at Time 1

		Threshold				Unique factor
Item description	Loading	$1	$2	$3	$4	
1. Spent a lot of time thinking about sex/ masturbation or planned sex?	0.87	− 0.93	− 0.25	0.63	1.34	0.23
2. Felt an urge to masturbate/have sex more and more?	0.92	− 0.7	− 0.09	0.69	1.38	0.16
3. Used sex/masturbation in order to forget about/escape from personal problems?	0.73	− 0.32	0.19	1.03	1.57	0.47
4. Tried to cut down on sex/masturbation without success?	0.7	0.32	0.74	1.31	1.79	0.51
5. Become restless or troubled if you have been prohibited from sex/masturbation?	0.77	0.24	0.74	1.41	1.95	0.41
6. Had so much sex that it has had a negative impact on your private relationships, economy, health or job, studies?	0.63	1.03	1.54	2.09	2.18	0.6

### Post Hoc Analysis of Temporal Stability of the BYSAS Latent Factor

Given support for longitudinal measurement invariance, we examined the post hoc analysis to assess the temporal stability of the BYSAS latent factor. This involved examination of the intercorrelations of Time 1, Time 2, and Time 3 latent factors of the model used to examine configural invariance. This model showed a good fit, χ^2^ = 266.89, *p* < 0.05; RMSEA = 0.05 (0.04–0.06); CFI = 0.98; TLI = 0.98. The correlations for Time 1 with Time 2 and Time 3 were 0.68 and 0.57, respectively; and the correlation of Time 2 and Time 3 was 0.61, They were all significant (*p* < 0.001) with large effect [36], there indicting strong temporal stability.

## Discussion

### Summary of Study Findings

Our study contributes novel insights into the longitudinal measurement and structural invariance of BYSAS items in adults over two years, spanning three-time points. At the measurement level, the findings demonstrated that there was support for configural invariance (same factor structure pattern), full loading invariance (consistent factor loadings), threshold invariance (consistent response level), and unique factor (same unique variances) invariance. At the structural level, there was support for latent variances (same latent variances) and latent covariances (same latent variances) invariance. Overall, these robust findings indicate strong support for longitudinal measurement and structural invariance in adults for BYSAS items across the specified time interval. Moreover, there was support for equivalency for latent mean scores across the three-time points. Although not related to invariance, we also found support for strong temporal stability for the latent factors across the three-time points.

### Meaning of our Invariance Findings

In the context of the current study, the support for configural invariance indicates that the same overall factor structure (one factor in the current study) holds across the three-time points. The support for loading invariance indicates that the strength of the associations of the items with the BYSAS latent factors is consistent for like items at all three-time points. Threshold invariance signifies that individuals endorsing the same observed scores exhibit equivalent latent trait scores across different time points. The support for unique latent factor invariance indicates that the reliabilities of the BYSAS items are the same for like items at all three-time points. Latent variances and covariances invariance indicate consistent variability in latent variables and their relationships across the three-time points. Notably, our study introduces a novel examination of longitudinal measurement and structural invariance in BYSAS item ratings, adding valuable knowledge to this field.

Two noteworthy findings, although not directly aligned with our primary objective of evaluating measurement, warrant attention. First, we found support for strong temporal stability for the latent factors across the three-time points, suggesting that sexual addiction has strong stability over time [17]. Second, prior to the measurement invariance analyses, we examined the fit for the one-factor BYSAS model at time points 1, 2, and 3. Overall, we interpreted our findings as showing mixed fit as the RMSEA showed unacceptable fit, especially at Time 2 and Time 3. Further examination of local misfit indicated that (residual and modification indices) showed a residual correlation of ≥ 0.10 between item 1 and item 6 at time 1; item 1 with items 2 and 6; and item 2 with items 5 and 6, and item 5 with item 6 at time 2; and items 3 and 6; and item 2 and item 6 at time 3. Thus, at each time point, the relevant items in these relations may not be represented well in the one-factor BYSAS models.

### Clinical, Practical, and Revision Implications

First, the strong support for longitudinal measurement and structural invariance in adults for the BYSAS items across three time points, spanning two years, suggests that the BYSAS scores remain free from biases related to scaling and measurement issues. Consequently, these scores can be reliably compared over the specified interval, allowing for accurate monitoring of developmental changes in sex addiction symptoms and assessment of clinical treatment effects over time. However, it’s essential to approach this recommendation with caution.

Second, strictly speaking, our findings pertain directly to the sex addiction symptoms encompassed by the BYSAS and not necessarily to sex addiction symptoms universally. Despite this, there are reasons to suspect that such a possibility cannot be ruled out. The BYSAS items draw from the components model of addiction [10, 13], designed to capture core addiction symptoms (salience/preoccupation, mood modification, tolerance, withdrawal symptoms, conflict, and relapse). Thus, it can be speculated that the findings in this present study could be relevant to other measures of sex addiction and potentially addiction in general. Specifically, the integration of our study’s findings with the recent developments in the classification of PSB, particularly with the acknowledgment of CSBD in the ICD-11, highlights the evolving understanding of sexual behavior disorders. Our findings, affirming the longitudinal measurement invariance of the BYSAS, not only support its reliability and validity over time but also reinforce the scale’s utility in both clinical and research settings amidst changing diagnostic landscapes. This robustness and adaptability of the BYSAS are essential for ongoing and future research into the nature, assessment, and treatment of sex addiction, providing a consistent and reliable tool against the backdrop of evolving clinical definitions and criteria. In light of these developments, our study contributes to the broader discourse on PSB, offering empirical evidence that supports the nuanced understanding and classification of these behaviors.

Third, our initial evaluation of the BYSAS one-factor model revealed some local misspecification, particularly with problems for item 6 across all three time-points. This item also showed high skewness and kurtosis values at all time points. Consequently, it can be speculated that it would be prudent for future studies to consider some revisions for the BYSAS, focusing particularly on item 6.

### Limitations

Although the current study has delivered original and valuable insights regarding the longitudinal measurement and structural invariance of the BYSAS symptom ratings across time, the findings and interpretations need to be considered with several limitations in mind. First, sex addiction ratings are influenced by age, gender, marital status, and education level [1]. Not controlling for these variables in the present study may have confounded findings. Second, the non-random selection of participants from the general community introduces additional confounding factors, limiting the generalizability of our findings, especially concerning individuals with clinically significant levels of sex addiction. Thirdly, reliance on self-rating questionnaires, such as the BYSAS, may have influenced the ratings and introduced common method variance, impacting the validity of our results. Fourthly, our findings have been obtained from a single study, and therefore replication is essential to validate the present findings. Fifth, while we establish sufficient power for the study, a larger sample size might yield different results. Additionally, a notable limitation related to item 6, as highlighted in the item response theory study by Zarate et al. [18], underscores its low reliability across the trait spectrum, coupled with low discrimination ability and high latent trait levels needed for endorsement. Given these limitations, future research should address these concerns to advance our understanding of longitudinal measurement and structural invariance in the context of sex addiction.

## Conclusions

In summary, the key finding in this present study is that BYSAS scores, assessed at different time intervals, are not confounded by biases related to scaling and measurement issues. Consequently, it can be reliably used to monitor developmental changes in sex addiction symptoms and assess the impacts of clinical treatment over time. Despite the limitations mentioned, the novelty of this study lies in being the first to investigate the longitudinal measurement invariance of self-ratings for BYSAS symptoms. This contribution holds significant promise for both theoretical advancements and practical implications in the field of sex addiction. We recommend that clinicians and researchers consider the findings and interpretations from this present study when integrating information on sex addiction symptoms obtained longitudinally across time.

## Electronic Supplementary Material

Below is the link to the electronic supplementary material.

Supplementary Material 1

## Data Availability

All data and syntax supporting the findings of this study are available with the article and its supplementary materials.
