# Supplementary material for: The Bergen–Yale Sexual Addiction Scale (BYSAS): Longitudinal Measurement Invariance Across a Two-Year Interval
Source: Psychiatr Q. 2024 Aug 29;95(4):561–77. doi: 10.1007/s11126-024-10087-6 (PMC11568058; doi:10.1007/s11126-024-10087-6)
Supplement: Supplementary file 1 — Supplementary Material 1 [file 11126_2024_10087_MOESM1_ESM.docx]

**Supplementary Table S1**

*Testing for Differences between Respondents and Nonrespondents at Time 3 for Hours Playing Video Game, and IGD Total Scores for Respondents and Nonrespondents at Time 1 and Time 2*

| Variable | Attrition | N | Mean | SD | *t* | *df* | *p* |
| --- | --- | --- | --- | --- | --- | --- | --- |
| Hours during weekdays playing video games: T1 | Respondent | 276 | 8.27 | 9.34 | 1.59 | 958 | .111 |
|  | Nonrespondent | 684 | 9.58 | 12.29 |  |  |  |
| Hours during weekends playing video games: T1 | Respondent | 275 | 6.34 | 4.96 | 1.43 | 957 | .152 |
|  | Nonrespondent | 684 | 6.93 | 6.02 |  |  |  |
| Hours during weekends playing video games: T2 | Respondent | 273 | 5.53 | 8.53 | 0.91 | 453 | .363 |
|  | Nonrespondent | 182 | 4.84 | 6.71 |  |  |  |
| Hours during weekends playing video games: T2 | Respondent | 274 | 6.04 | 4.87 | 0.08 | 452 | .937 |
|  | Nonrespondent | 180 | 6.08 | 5.62 |  |  |  |
| IGD total: T1 | Respondent | 273 | 6.33 | 0.30 | 1.18 | 962 | .239 |
|  | Nonrespondent | 691 | 6.76 | 0.19 |  |  |  |
| IGD total: T2 | Respondent | 459 | 6.53 | 5.03 | 0.60 | 962 | .549 |
|  | Nonrespondent | 505 | 6.73 | 6.06 |  |  |  |

**Supplementary Table S2**

*Descriptive and Dispersion Statistics for the DYSAS items at Times 1, 2 and 3*

| Items | Valid | Missing | Mean | *SD* | Skewness | *SE* of Skewness | Kurtosis | *SE* of Kurtosis | Shapiro-Wilk |
| --- | --- | --- | --- | --- | --- | --- | --- | --- | --- |
| Time 1 | | | | | | | | | |
| BYSAS1 | 276 | 0 | 1.78 | 1.19 | 0.13 | 0.15 | -0.78 | 0.29 | 0.91*** |
| BYSAS2 | 276 | 0 | 1.62 | 1.25 | 0.24 | 0.15 | -0.92 | 0.29 | 0.90*** |
| BYSAS3 | 275 | 1 | 1.26 | 1.22 | 0.58 | 0.15 | -0.63 | 0.29 | 0.85*** |
| BYSAS4 | 275 | 1 | 0.73 | 1.12 | 1.42 | 0.15 | 1.01 | 0.29 | 0.69*** |
| BYSAS5 | 275 | 1 | 0.74 | 1.06 | 1.33 | 0.15 | 0.89 | 0.29 | 0.72*** |
| BYSAS6 | 276 | 0 | 0.25 | 0.69 | 3.46 | 0.15 | 13.35 | 0.29 | 0.41*** |
| Time 2 | | | | | | | | | |
| BYSAS1 | 276 | 0 | 1.70 | 1.22 | 0.16 | 0.15 | -0.81 | 0.29 | 0.90*** |
| BYSAS2 | 276 | 0 | 1.57 | 1.21 | 0.27 | 0.15 | -0.83 | 0.29 | 0.90*** |
| BYSAS3 | 276 | 0 | 1.14 | 1.22 | 0.79 | 0.15 | -0.38 | 0.29 | 0.83*** |
| BYSAS4 | 276 | 0 | 0.59 | 1.00 | 1.79 | 0.15 | 2.62 | 0.29 | 0.65*** |
| BYSAS5 | 276 | 0 | 0.65 | 1.00 | 1.53 | 0.15 | 1.67 | 0.29 | 0.69*** |
| BYSAS6 | 276 | 0 | 0.22 | 0.66 | 3.68 | 0.15 | 14.71 | 0.29 | 0.38*** |
| Time 3 | | | | | | | | | |
| BYSAS1 | 276 | 0 | 1.66 | 1.13 | 0.2 | 0.15 | -0.6 | 0.29 | 0.91*** |
| BYSAS2 | 276 | 0 | 1.45 | 1.21 | 0.37 | 0.15 | -0.81 | 0.29 | 0.88*** |
| BYSAS3 | 276 | 0 | 1.12 | 1.24 | 0.81 | 0.15 | -0.38 | 0.29 | 0.82*** |
| BYSAS4 | 276 | 0 | 0.64 | 1.03 | 1.64 | 0.15 | 1.99 | 0.29 | 0.67*** |
| BYSAS5 | 276 | 0 | 0.65 | 1.03 | 1.71 | 0.15 | 2.39 | 0.29 | 0.68*** |
| BYSAS6 | 276 | 0 | 0.17 | 0.49 | 3.84 | 0.15 | 19.55 | 0.29 | 0.39*** |

*Note*. *SD* = standard deviation; *SE* = standard error.

**Supplementary Table S3**

*Brief Description of the Parameterization of the Configural, Loading, Threshold and Unique Factor Invariance Models*

In the baseline or configural invariance model (Model 1), constraints were imposed on this model for model identification. They included (1) fixing all intercepts at 0 at each time point; (2) at time 1, fixing the common factor mean to zero, and for all other time points, freely estimating, the unique factor covariance matrix; (4) the same observed indicator is chosen as the marker variable for all time points, and the factor loading of the marker variable is constrained to be 1.00; (5) one threshold for each indicator (and a second threshold for the marker variable) is constrained to be equal across all time points; and (6) the loadings for the first items in the factor model at each time point were set to one to identify the scales of the latent factors. The baseline model should show good fit to infer support for configural invariance and also to continue evaluation of the loading, threshold, and unique factor invariance models.

Following support for this this, the loading invariance model was tested by constraining the factor loadings to be are identical across time. Support for this model is inferred if this model does not differ from model 1. With support for Model 2, the threshold invariance model I tested that tests by for constraining the thresholds to be are identical across time. Support for this model is inferred if this model does not differ from model 2. When there is support, the support for unique factor invariance model (Model 4) is examined by adding the constraint that corresponding unique factor variances are equal over time (the lagged unique factor covariances over time are freely estimated), and this model is compared to model 3. Support for this model is inferred if this model does not differ from model 3

It is important to note that for measured ordered-categorical indicators having invariant factor loadings and invariant thresholds does imply that changes in the means of the measured ordered-categorical indicators can be solely attributed to changes in the latent common factor. To achieve this, unique factor invariance must be reached.

**Supplementary Figure S1**

Path Diagram of the 1-Factor Model for the BYSAS Items for Evaluating Longitudinal Measurement Invariance


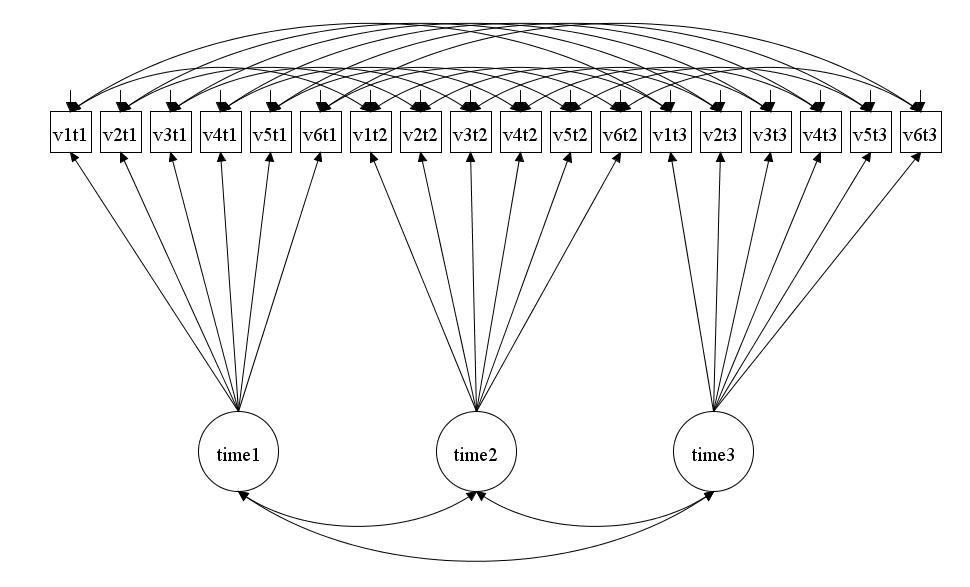


*Note*. The residuals are not shown in the figure. V1t1 to v6t1 are items for time1; v1t2 to v6t2 are items for time 2; and v1t3 to v6t3 are items for time 3.
